# A protocol for cloning, expression, and purification of Lysine exporter (LysE) gene of Mycobacterium tuberculosis

**DOI:** 10.12688/f1000research.131768.3

**Published:** 2024-02-05

**Authors:** Shilpa Upadhyay, Archana Dhok, Vinod Agarkar, Supriya Kashikar, Zahiruddin Syed Quazi

**Affiliations:** 1 Research Associate, Division of Evidence Synthesis, Global Evidence Synthesis Initative (GESI), Datta Meghe Institute of Higher Education and Research, Wardha, Maharashtra, 442001, India; 2 Professor and Head, i-Health Consortium, Department of Biochemistry, Jawaharlal Nehru Medical College, Datta Meghe Institute of Higher Education and Research, Wardha, Maharashtra, 442004, India; 3 Director, Research and Development, GeNext Genomics Private Limited, Nagpur, Maharashtra, 440010, India; 4 CEO, GeNext Genomics Private Limited, Nagpur, Maharashtra, 440010, India; 5 Director, One Health Centre, Global Consortium for Public Health and Research (GCPHR), Department of Community Medicine, Jawaharlal Nehru Medical College, Datta Meghe Institute of Higher Education and Research, Wardha, Maharashtra, 442004, India

**Keywords:** Lysine exporter (LysE), Amino acid transporter, Anti-tubercular agent, Expression and purification, Immobilized Metal-Affinity Chromatography (IMAC), Histidine-tag, Bacterial proteins, Tuberculosis

## Abstract

**Background:**

Tuberculosis (TB) is among the deadliest diseases and a significant cause of illnessacross the globe. Several studies on mycobacterial proteins, such as proteases and transporters that are essential for survival and pathogenesis have aimed to develop an efficient anti-tubercular agent. In mycobacterium, lysine exporter (LysE) is an amino acid transporter and a probable target for an anti-tubercular agent as it is responsible for bacterial growth inhibition and is also absent in the widely used Bacillus Calmette-Guérin (BCG) vaccine.

**Methods:**

Some studies have purified LysE using different protocols. This study describes a protocol for purifying different constructs of LysE, focusing on its hydrophobic region using immobilized metal affinity chromatography (IMAC) after expressing LysE gene in a bacterial expression system. pET vector (pET28a) is used as an expression vector. Amplified LysE gene is ligated with the pET28a vector, and the resultant plasmid is then transformed into E. coli cells. The vector has a histidine tag that makes the purification process convenient. After IMAC, the samples will be subjected to size-exclusion chromatography for further purification.

**Results:**

Cloning and amplification findings will be analyzed using 1% agarose gel, and protein expression and purification outcomes will be examined using sodium dodecyl-sulfate polyacrylamide gel electrophoresis (SDS-PAGE). Domain-specific constructs of LysE can be further analyzed as an anti-tubercular agent.

**Conclusions:**

Despite being a potential anti-tubercular target, research is quite limited on this protein. Therefore, we aim to purify LysE protein for further analysis. Similar protocols have already been implemented to purify several other bacterial proteins with >95% purity.

## Introduction

Tuberculosis (TB) is caused by *Mycobacterium tuberculosis (M.tb)*, a slow-growing bacteria and a very successful pathogen that acclimates to staying alive inside the host ( Chai *et al.*, 2018). According to the World Health Organisation (WHO), 1.5 million people globally died from TB in 2020 ( WHO key facts, 2021). In the current scenario, the Bacillus Calmette-Guérin (BCG) vaccine is the only available TB vaccination that is widely used and protects newborns and children from TB meningitis. However, BCG’s significance in reducing tuberculosis transmission is restricted since it is considered less efficient in preventing pulmonary TB ( Pereira *et al.*, 2007). As a result, it is essential to investigate the M. TB proteins lacking in BCG and capable of eliciting particular humoral responses, cell-mediated responses, and appropriate innate responses in the host ( Chen, 2018).

In bacteria, L-Lysine exporters are transmembrane proteins that remove the surplus of metabolically produced L-lysine and L-arginine from the cytosol. Its deficiency causes high L-lysine cellular levels, inhibiting bacterial growth ( Georgieva *et al.*, 2020a). In this protocol, we intend to clone, express, and purify LysE using immobilized metal affinity chromatography (IMAC) to yield protein with high concentration and >95% purity. If the protein is secretory, it is secreted to the cell supernatant and purified by IMAC chromatography using a nickel-nitrilotriacetic acid (Ni-NTA) agarose column. If the protein is present in inclusion bodies (insoluble form), then an extraction buffer containing urea will be used, followed by Ni-NTA purification. Similar protocols have already been adapted to purify other mycobacterial proteins in our collaborative lab with >95% purity.

## Methods

### Instruments required

1. For cloning: Thermal cycler PCR machine (Eppendorf), electrophoresis assembly (Bio-Rad), gel doc (Vilber), incubator shaker (Labbyscopes), plasmid isolation kit and Gel/PCR extraction kit ((BR Biochem), laminar airflow, water bath (REMI), UV illuminator (Biotech), -80°C and -20°C refrigerator, PCR tubes, Milli-Q Reference Water Purification System (Merck Millipore)
2. For expression and purification: Sonicator (Lobalite), weighing balance (Contech), spectrophotometer (Elico), Cooling centrifuge (REMI), pH meter (BR Biochem), autoclave, dialysis tube, Hot air oven (Sentwin), protein concentrator (Millipore), syringe filter, vortex, falcons, Petri plates, pipettes, vials, tips, beakers, conical flasks, ice flaker, electrophoretic assembly (Bio-Rad), Superdex S200 16/60 columns, AKTA purifier 100.

### Reagents required

M.tb LJ slants, E. coli strains (DH5α, BL-21 (DE3)), Primers (forward and reverse), restriction enzymes for cloning and amplification from New England Biolabs, Plasmids and bacterial strains from Novagen, and Ni-NTA resin for purification from Qiagen (Valencia, CA) will be purchased. Buffers and other chemicals from SIGMA, MP Biomedicals, Invitrogen, Amresco, Himedia, BR-Biochem etc.

1. **For genomic DNA isolation**: Sodium glutamate, Tris-HCl, EDTA, Lysozyme, RNAse A, Proteinase K, phenol, chloroform, Isoamyl alcohol, sodium acetate, isopropanol, ethanol, agarose, boric acid, tween-80, NaCl, Ethidium Bromide (EtBr).
2. **For competent cells preparation**: E. coli strain ( *DH5α*, *BL-21* and *Rosetta-2*), Luria-Bertani (LB) broth, Polyethylene glycol (PEG) 3350, Dimethyl sulfoxide (DMSO), MgSO _4_, transformation and storage (TSS) buffer, ice, liquid nitrogen.
3. **For cloning into pET expression vector**: Restriction enzymes, pET vectors, T4 DNA ligase, Taq DNA polymerase, forward and reverse primers, agarose, competent cells, antibiotics (kanamycin and chloramphenicol), nuclease-free water, MgCl _2_, Deoxynucleotide triphosphates **(**dNTPs), Bovine Serum Albumin (BSA), Sodium borate or tris borate buffer, Ethidium Bromide (EtBr), Glycerol, Agarose, ice, DNA ladder.
4. **For transformation**: LB agar media and broth, antibiotics, comp cells, 5X KCM (KCl, MgCl _2_, CaCl _2_), nuclease-free water, agar plates, and ice.
5. **For protein expression and purification**: LB broth, antibiotics, glycerol, Isopropyl β-d-1-thiogalactopyranoside (IPTG), ice, Urea, Sodium Dodecyl Sulphate (SDS), Nickel- Nitrilotriacetic acid (Ni-NTA) resin, Tris, Sodium Chloride (NaCl), β-mercaptoethanol, phenylmethylsulfonyl fluoride (PMSF), imidazole, Mili Q water, HCl, ethanol, acrylamide, bis-acrylamide, ammonium persulphate, tetramethylethylenediamine (TEMED), dithiothreitol (DTT), glycine, bromophenol blue, protein marker, coomassie brilliant blue, methanol, glacial acetic acid, Bradford’s reagent, bovine serum albumin (BSA), phosphoric acid, DNA and protein ladder, Coomassie Brilliant Blue R-250.

### Protocol

**Genomic DNA isolation**

Isolation and purification of genomic DNA using mycobacterium tuberculosis protocols was performed according to the earlier protocols ( Van Helden *et al.*, 2001). Briefly, heat the sealed culture tube of M. tuberculosis H37Rv at 80°C for one hour. Perform all steps in the Class III biosafety cabinet for safety measures. Add 3 ml of extraction buffer (50 mM Tris-HCl, 25 mM EDTA, 5% monosodium glutamate, pH 7.4) in LJ slant and scrape the bacterial colonies using a disposable loop. Collect the buffer and bacterial colonies in a 50 mL falcon tube containing 30 glass beads of 5mm diameter. Again add 3 ml extraction buffer in LJ slant to collect the remaining colonies. Disrupt the suspension by vortexing the tightly sealed falcon at full speed for 2 to 3 minutes to ensure no clumps are left. Lyse the cells using 400 μL of 50 mg/mL lysozyme and add 10 μL of 10 mg/mL RNAase for RNA degradation, followed by incubation for 2 hours at 37°C. Add 600 μL of 10× proteinase K buffer (100 mM Tris-HCl, 50 mM EDTA, 5% SDS, pH 7.8). Add 150 μL of 10 mg/mL proteinase K to remove protein contamination, mix and incubate at 45°C for 16 h. Perform DNA purification by adding 5 ml of phenol, chloroform, and isoamyl alcohol (25:24:1) and slowly mixing by inverting tubes four to five times. Throughout 30 minutes, redo the inversion steps four times. Gently remove the supernatant after centrifuging at 3000g for 20 minutes at room temperature. Add 5 mL of chloroform/isoamyl alcohol (24:1) again, gently mix, and centrifuge at 3000g for 20 minutes at room temperature. Add 700 μl of 3 M sodium acetate, pH 5.5, to the supernatant that has been collected. Add the same amount of isopropanol and mix gently by inverting the tube two to four times. Use a glass rod or sealed pipet tip to collect the DNA, or incubate at -20°C for 30 minutes and then centrifuge it for 30 minutes at 3000g. Wash the pellet in 5 mL 70% ethanol and air dry at 55°C until dry. Finally, suspend the alcohol-precipitated dried *M. tuberculosis* genomic DNA in 500 μL of lukewarm 1× TE buffer and store it at -80°C. Check the isolated genomic DNA in 1% agarose gel ( Van Helden *et al.*, 2001).

**Composition of 1× TE buffer**

10 mM Tris-HCl, 1 mM EDTA, pH 8.0.

### Preparation of competent cells

Inoculate 10 μl glycerol stock of E. coli strain ( *DH5α*, *BL-21 DE3* and *Rosetta-2*) in 10 ml LB broth (without antibiotics for *DH5α* and *BL-21* and 2 μl chloramphenicol for *Rosetta-2*), and incubate overnight at 37°C. The next day inoculate 1 ml overnight culture in 100 ml LB broth (without antibiotic for *DH5α* and *BL-21* and 20 μl chloramphenicol for *Rosetta-2*). Allow it to grow at 37°C until O.D. comes to 0.35-0.4. Swirl the culture flask on ice for 1-2 minutes to pre-chill the cells. Pellet down the cells at 5000 rpm for 10 min at 4°C. Resuspend in 10 ml ice-cold TSS buffer (10 ml TSS for 100 ml culture pellet). Aliquots 50 μl in prechilled vials and snap freeze in liquid nitrogen and store at -80°C.

**Preparation of LB broth**:

Weigh 25 g LB media and dissolve it in 1 liter of distilled water.

**Composition of TSS buffer:**

TSS buffer: 100 ml

**LB**: 73 ml

**10% PEG 3350**: 20 ml (from 50% stock)

**5% DMSO**: 5 ml

**20 mM MgSO**_**4**_: 2 ml (from 1 M stock)

### Amplification of gene by using specifically designed primers, cloning in a suitable expression system

**Construction of recombinant plasmid**

After retrieving the gene sequence of LysE (Rv1986) from the NCBI nucleotide database, design gene-specific forward primer 5′-ATGAACTCACCACTGGTCGTCGG-3′ and reverse primer 5′-CTAGGTCACGGTCAGCGAGATTCC-3′ to amplify the gene of interest isolated from the genomic DNA of Mycobacterium tuberculosis using PCR. Check on 1% agarose gel.

Design four different constructs from the LysE gene sequence (Full length, V6, A11, and A64). Perform second PCR using amplified gene from 1 ^st^ PCR as a DNA template and forward primers *Nde1 (CATATG)* and *BamH1 (GGATCC)* restriction site inserted in the four different forward primers

1. **N2_Nde1 FP1**: 5′-TAGTGCTA ***CATATG*** AACTCACCACTGGTCGTCGG-3′
2. **V6_BamH1 FP1:** 5′-AATTTT ***GGATCC*** GTCGTCGGCTTCCTGGCCTGC-3′
3. **A11_BamH1 FP1:** 5′-TTAATT ***GGATCC*** GCCTGCTTCACGCTGATCG-3′
4. **A64_Nde1 FP1:** 5′-TAATTTA ***CATATG*** GCACATCCGCGTGCGCTCAATGTCG-3′

and *Xho1 (CTCGAG **)*** restriction site inserted in reverse primer 5′-ATTTT ***CTCGAG*** CTAGGTCACGGTCAGCGAGATTCC-3′, respectively. The reverse primer remains the same for all four constructs.

Analyze the amplified product on 1% agarose gel electrophoresis. Cut and elute from the gel, clean up using the Gel/PCR recovery kit (Zymoclean), and proceed for digestion. See Table 1 for the PCR mixture.

**Table 1.** PCR mixture for amplification.

Sr. No.	PCR components	Volume	Concentration
1	Taq polymerase (1 unit/μl)	1.0 μl	1 unit
2	10× PCR buffer	2.0 μl	1×
3	MgCl _2_ (50 mM)	0.6 μl	1.5 mM
4	dNTPs (10 μM)	0.4 μl	200 pM
5	Autoclaved Distilled water	12 μl	
6	Primer I (4 μM) (forward primer)	1.0 μl	0.2 μM
7	Primer II (4 μM) (reverse primer)	1.0 μl	0.2 μM
8	Template DNA	2.0 μl	10-20 ng

**PCR conditions:**

Denaturation: 95°C (1 min)

Annealing: 57°C for 1 ^st^ PCR and 64-66°C for 2 ^nd^ PCR (1.30 min)

Extension: 72°C (2 min)

Number of cycles: 30

**Expression vector (pET28a) preparation:**

The expression vector (pET 28a) will be provided by our collaborative lab (GeNext Genomics Pvt. Ltd.). Grow 10 ml culture of pET28a vector overnight at 37°C. The next day, isolate the plasmid using a plasmid isolation kit.

**Digestion**

**Double digestion of vector:** 100 μl reaction volume

Digest the expression vectors using *Nde1, Xho1* enzymes, and *BamH1* and *Xho 1* restriction enzymes.

Incubate overnight at 37°C. Analyze the digested product on 1% agarose gel electrophoresis. Cut and elute from the gel and clean up using the Gel/PCR recovery kit (Zymoclean). See Table 2 for double digestion mixture for vector.

**Table 2.** Vector double digestion reaction mixture.

S.No	Components	Volume
**1.**	H _2_O	5 μl
**2.**	Restriction enzyme buffer	10 μl
**3.**	BSA	1 μl
**4.**	Vector	80 μl
**5.**	*Nde I/BamH1*	2 μl
**6.**	*Xho I*	2 μl

**Double digestion of amplified LysE:** 50 μl reaction volume

Digest the amplified LysE using *Nde1/BamH1* and *Xho1* enzymes. Incubate overnight at 37°C. The next day, analyze and purify from 1% agarose gel electrophoresis using Gel/PCR recovery kit. See Table 3 for amplified LysE double digestion mixture.

**Table 3.** Amplified product double digestion reaction mixture.

S.No	Components	Volume
**1.**	H _2_O	17.5 μl
**2.**	Restriction enzyme buffer	5 μl
**3.**	BSA	0.5 μl
**4.**	Amplified LysE	25 μl
**5.**	*Nde I/BamH1*	1 μl
**6.**	*Xho I*	1 μl

**Ligation**

Ligate the digested gene and vector using *T4 DNA ligase* and transform it into chemically competent E. coli *DH5α* cells. See Table 4 for ligation reaction mixture.

**Table 4.** Ligation reaction mixture.

S.No	Components	Volume
**1.**	Vector	1 μl
**2.**	Insert	1 μl
**3.**	*T4 DNA Ligase*	1 μl
**4.**	Buffer	10 μl
**5.**	H _2_O	7 μl

**Transformation**

Prepare LB agar plates with antibiotic selection. Take 50 μl *DH5α* competent cells + 20 μl 5X KCM + 5 μl ligation mixture + 25 μl H _2_O. Add all the above components, mix well, and keep it on ice for 20 mins. Heat shock at 37°C for 5 mins. Add 500 μl LB media and incubate at 37°C on the shaker for one hour. Centrifuge at 8000 rpm for 3 mins. Discard 550 μl supernatant and resuspend the pellet in the remaining 50 μl supernatant.

Plate the transformed cells on Luria–Bertani (LB) agar plates with antibiotics (kanamycin 50 μg/ml) which act as a selection marker for the pET 28a vector, and incubate overnight at 37°C. The next day, screen the positive clones with gene-specific primers and restriction digestions.

**Composition of 5× KCM buffer (10 ml)**

2M KCl = 2.5 ml

1M MgCl _2_ = 1.5 ml

1M CaCl _2_ = 2.5 ml

Mili Q water = 3.5 ml

**Colony PCR:**

Initiate independent PCR reactions for each different primer pair. Mark colonies on a plate, take 20 μl of water in separate tubes, touch colonies from the tip, and dissolve them in 20 μl of water in respective tubes. Prepare glycerol stock by adding 10 μl of the above cells in 100 μl of LB broth with the required antibiotic before disruption. Vortex it properly to disrupt the cells. Take PCR tubes, mark accordingly, and transfer 1 μl from 20 μl dissolved cells in a PCR tube. Add 500 μl LB to the remaining 19 μl cells, and allow it to grow for 1 hr at 37°C, 200 rpm. Check on 1% agarose gel after PCR. Following confirmation of the positive clone, take 10 ml LB + kanamycin add 100 ul of selected clone (grown for 1 hr), and allow it to grow overnight at 37°C, 200 rpm. The next day, isolate the plasmid using a plasmid isolation kit and transform it into BL21 (DE3) competent cells. See Table 5 for colony PCR reaction mixture.

**Table 5.** Colony PCR reaction mixture.

Sr. No.	PCR components	Volume	Concentration
1	Taq polymerase (1 unit/μl)	1.0 μl	1 unit
2	10× PCR buffer	2.0 μl	1×
3	MgCl _2_ (50 mM)	0.6 μl	1.5 mM
4	dNTPs (10μM)	0.4 μl	200 pM
5	Autoclaved Distilled water	12 μl	
6	Primer I (4 μM) (forward primer)	1.0 μl	0.2 μM
7	Primer II (4 μM) (reverse primer)	1.0 μl	0.2 μM
8	Template DNA	2.0 μl	10-20 ng

**Protein expression and purification**

Transform LysEpET28a plasmid in BL21 (DE3) competent cells and inoculate in LB growth media supplemented with kanamycin (50 μg/ml) and allow it to grow overnight at 37 ^o^C. Re-inoculate the overnight grown culture in flasks containing LB media with antibiotics and allow it to grow for three hours at 37°C at 220 rpm until the O.D. comes to 0.6 to 0.8. Transfer half of the culture at 18°C and after 30 minutes, induce both (18°C and 37°C) cultures using (0.1 to 1 mM) isopropyl β-D-1-thiogalactopyranoside (IPTG) (take out uninduced sample).

Harvest the 37°C culture after 3 to 4 hours and the 18°C culture after 18-20 hours. Sonicate the harvested cells on ice in cold extraction **buffer A** containing 50 mM Tris-HCl pH 8.0, 300-600 mM NaCl, 20% glycerol, 10 mM βME, and (0.1 to 1 mM) PMSF. Centrifuge the sonicated cells for 30 minutes at 4°C at 16000 rpm. Collect the supernatant and store it at -80°C. Collect samples from each step.

For insoluble inclusion bodies, resuspend the remaining cell pellet at 37°C culture in an extraction buffer containing 0.1% Triton X-100. Shake gently at room temperature for 30-40 minutes and centrifuge at 16000 rpm for 20 minutes. Wash the resultant pellet twice with 50 mM Tris-HCl pH 8.0 and 500 mM NaCl buffer. Finally, separate the inclusion bodies from suspension by centrifugation at 16000 rpm and suspend them by mixing in extraction **buffer B** containing 50 mM Tris-HCl pH 8.0, 100-300 mM NaCl, 10-20% glycerol, 10 mM βME, and 6 M Urea. Analyze the presence of expressed protein on an SDS-PAGE gel.

**Protein purification**

*For soluble protein*

Perform complete purification steps in cold conditions. Centrifuge the solubilized cell pellet at 16,000 rpm for 30 minutes to remove cell debris. Meanwhile, wash the resin with water and equilibrate with **wash buffers** containing 50 mM Tris-HCl pH 8.0, 100-300 mM NaCl, and 10-20% glycerol and 5 mM. Wash again with the same buffer containing 20 mM imidazole. Allow the clear supernatant (that shows induction on SDS-PAGE gel) to bind with equilibrated Ni-NTA resin (for overnight rotation). Pack protein-bound resin in the column. Wash the protein-bound resin with wash buffers containing 5 mM and 20 mM imidazole. Finally, elute the bound protein with an **elution buffer** containing 300 mM imidazole. Collect the fractions at each step and run on an SDS-PAGE gel. Subject the eluted fractions for refolding (dialysis) to yield active protein in buffer containing 30 mM Tris HCl pH 8.0, 150 mM NaCl, 1 mM DTT, 1 mM EDTA, and 10% glycerol.

**For insoluble protein**

Perform complete purification steps in cold conditions. Centrifuge the solubilized cell pellet (inclusion bodies) at 16,000 rpm for 30 minutes to remove cell debris. Meanwhile, wash the resin with water and equilibrate with **wash buffers** containing 50 mM Tris-HCl pH 8.0, 100-300 mM NaCl, 10-20% glycerol, 5 mM and 6M urea. Wash again with same buffer containing 20 mM imidazole and 6M urea. Allow the clarified supernatant (that shows induction on SDS-PAGE gel) to bind with equilibrated Ni-NTA resin (for overnight rotation).

Pack protein-bound resin in the column. Wash the protein-bound resin with wash buffers containing 5 mM and 20 mM imidazole and Urea. Last wash will be of 50 mM Tris-HCl pH 8.0, 300 mM NaCl, 20% glycerol, 10 mM βME, and 6 M Urea and then followed by elution with the same buffer containing 300 mM Imidazole. Collect the fractions at each step and run on an SDS-PAGE gel. Subject the eluted fractions for refolding (dialysis) to yield active protein in buffer containing 30 mM Tris HCl pH 8.0, 150 mM NaCl, 1 mM DTT, 1 mM EDTA, 10% glycerol and 0.1 to 1 mM CaCl _2_ or MgCl _2_.

Proceed with the refolded protein for size exclusion chromatography (Superdex S200 16/60 GE Healthcare) attached with AKTA purifier 100. Equilibration and elution buffer consisting of 30 mM Tris-HCl pH 8.0, 300 mM NaCl, 2% glyxcerol, and 1 mM DTT. Collect the fractions and analyze them by SDS-PAGE ( Umbarkar *et al.*, 2019). Concentrate the purified protein using a molecular weight cut-off concentrator and measure the concentration by Bradford’s assay.

Protocol may change according to protein behavior during purification and stability.

**Size exclusion chromatography**

Proceed with the refolded protein for size exclusion chromatography (SEC). Superdex S200 16/60 column from GE Healthcare attached with AKTA purifier 100 assembly is used for the purification process. Equilibrate the column and loop before loading the sample in the column. Arrange vials in the collector assembly for fractions collection. Set the chromatographic conditions and inject the sample. Elute the fractions using elution a buffer consisting of 30 mM Tris-HCl pH 8.0, 300 mM NaCl, 2% glycerol, 1 mM DTT and 0.1 to 1 mM CaCl _2_ or MgCl _2_. The same buffer is used for equilibration. Collect the fractions (that shows peak) and analyze them by SDS-PAGE ( Umbarkar *et al.*, 2019). Concentrate the purified protein using a molecular weight cut-off concentrator and measure the concentration by Bradford’s assay.

**Chromatographic conditions:**

1. Flow rate: 1 to 1.2 ml/min
2. 0.1 to 0.3 MPa
3. Wavelength: 280 and 254 nm
4. Injection volume: 0.5 to 5 ml (depending on loop’s length)

Protocol may need to be changed according to protein behavior during purification and stability.

### Buffers

**Buffer A for expression check and sonication:** 50 mM Tris-HCl pH 8.0, 300-600 mM NaCl, 20% glycerol, 10 mM βME, and 0.1 to 1 mM PMSF.

**Extraction buffer (for native protein) Buffer C**: 50 mM Tris-HCl pH 8.0, 100-300 mM NaCl, 10-20% glycerol, 10 mM βME.

**Extraction buffer (for insoluble protein) Buffer B:** 50 mM Tris-HCl pH 8.0, 100-300 mM NaCl, 10-20 % glycerol, 10 mM βME, 6M Urea.

**Wash buffer A:** 50 mM Tris-HCl pH 8.0, 300 mM NaCl, 20% glycerol, 5 mM imidazole (6 M Urea for insoluble protein).

**Wash buffer B:** 50 mM Tris-HCl pH 8.0, 300 mM NaCl, 20% glycerol, 20 mM imidazole, (6 M Urea for insoluble protein).

**Elution buffer:** 50 mM Tris-HCl pH 8.0, 300 mM NaCl, 20% glycerol, 300 mM imidazole, (6 M Urea for insoluble protein).

**Dialysis buffer:** 30 mM Tris HCl pH 8.0, 150 mM NaCl, 1 mM DTT, 1 mM EDTA, and 10% glycerol.

**Buffer for size exclusion chromatography:** 30 mM Tris-HCl pH 8.0, 300 mM NaCl, 2% Glycerol, 1 mM DTT and 0.1 to 1 mM CaCl _2_ or MgCl _2_.

### Dissemination

Related studies will be published in indexed journals, and papers will be presented at relevant conferences.

### Study status

The study is ongoing. We are validating the protocol using previously purified genomic DNA. Primers, enzymes, reagents and instruments have been arranged.

## Discussion

L-lysine exporter (LysE) is a small transmembrane protein of 20-25 kDa that functions as a dimer and has five to six transmembrane hydrophobic spanning helices ( Eggeling and Sahm, 2001; Krämer, 2002). In bacteria, L-lysine exporters (transmembrane lysine exporters) remove the surplus of metabolically produced L-lysine and L arginine from the cytosol. Its deficiency causes high L-lysine cellular levels, inhibiting bacterial growth ( Georgieva *et al.*, 2020a). To remove the excess lysine from the cytosol, lysine exporters are present on the inner membrane of the bacterial cell. LysE is a target for bacterial inhibition since its deficiency leads to the accumulation of toxic levels or toxic analogs of L-lysine and then the inhibition of bacterial growth ( Georgieva *et al.*, 2020b).

LysE was identified in a few studies as a vaccine candidate, drug target, and a potential diagnostic marker ( Chen, 2018; Cockle *et al.*, 2002; Georgieva *et al.*, 2020a; Gideon *et al.*, 2010). LysE, also known as the Rv1986 gene in mycobacterium tuberculosis, stimulates innate and adaptive immune cell proliferation and cytokine release. These properties of mycobacterial LysE attract our attention to work and explore more on this protein.

Some studies have purified LysE protein, such as Georgieva *et al.* (2020a) purified LysE protein on lipodiscs made of native E. coli membranes and in detergent and further characterized it biophysically. Another study ( Chen, 2018) claimed to purify LysE as a full-length protein. However, the characterization of this protein is still a new area to explore. This protocol has been designed to purify the LysE gene in four different constructs considering its hydrophobic region using IMAC to unveil new knowledge about this protein. IMAC is a widely adopted method for purifying recombinant proteins containing a 6X histidine tag. In this protocol, we used Ni-NTA resin. Since the protein has a histidine tag, the imidazole ring of histidine readily forms coordination bonds with the immobilized nickel metal. Proteins with polyhistidine sequences can be easily eluted from the column by adding free imidazole to the elution buffer. This method has been developed with discussion with protein experts. Also, the procedures mentioned here have already been validated and implemented in purifying other mycobacterial proteins with >95% purity in our collaborative lab ( Umbarkar *et al.*, 2019).

### Additional Information

From 1 L culture, we were able to purify 10-15 mg of similar type of mycobacterial protein. This yield depends upon the expression of protein depending on type of construct, amino acids sequence, extra tags, folding nature of protein, expression time, temperature and concentration of IPTG etc.

Once we are able to purify recombinant LysE protein, SDS gels containing expression and purification work will be published in further publications.

### Note

1. While performing experiments, the buffer’s composition and experimental conditions may slightly differ from the composition and conditions mentioned above depending on the protein’s behavior and stability (secretory or insoluble).
2. The concentration of IPTG, urea, PMSF, glycerol, and imidazole may differ after the analysis of expressed protein on SDS-PAGE.
3. To purify protein with high purity (>95%), we are adopting the following steps. Which we will publish in details later on getting recombinant protein.
  • First step: As we made the construct in such a way so that protein yield would get as much as at increased level.
  • Second step: The interacting proteins from protein expressing strain with target protein should be as low as possible. However, placing 6 His tag with target protein minimized the problem of protein binding column.
  • Third step: Buffers and their stock should be prepared in autoclaved water and stored after filtration.
4. This protocol follows the principles of Good Laboratory Practice (GLP).

### Troubleshooting tips

1. During Ni-NTA purification, sometimes, while adding the supernatant containing extraction buffer (50 m MTris-HCl pH 8.0, 100-300 mM NaCl, 10-20% glycerol, 10 mM βME), the color of the resin may change from blue to brown. This is due to the presence of β-mercaptoethanol (reducing agent) in extraction buffer. This brown color of resin will disappear after 3-4 washing with wash buffer A containing no βME. This color change will not affect the purification step.
2. During dialysis or refolding of insoluble protein, sometimes protein may get precipitate. To avoid the precipitation, step wise dialysis is required. Since the elution buffer for insoluble protein contains 6 M or 8 M urea. Step wise removal of urea is recommended. For e.g., use dialysis buffer 1 with 4 M urea, dialysis buffer 2 with 2 M urea, dialysis buffer 3 with 1 M urea and then the final dialysis buffer with no urea.
3. Concentration of Urea (6 M to 8 M) may change depending of the nature of protein.

### Ethical consideration

This work has been approved by the Institutional Ethics Committee (IEC), Datta Meghe Institute of Higher Education and Research, Wardha, Maharashtra.

## Data Availability

No data are associated with this article.
