# Supplementary material for: A protocol for cloning, expression, and purification of Lysine exporter (LysE) gene of Mycobacterium tuberculosis
Source: F1000Res. 2024 Feb 5;12:297. Originally published 2023 Mar 17. [Version 3] doi: 10.12688/f1000research.131768.3 (PMC10811417; doi:10.12688/f1000research.131768.3)
Supplement: Peer review history

# Reviewer report: Reviewer response for version 2

**DOI:** 10.5256/f1000research.150461.r232751

**Published:** 2024-01-25

**Authors:** Dr. Rahul Sharma

**Affiliations:** 1 University of Virginia, Virginia, USA

The protocol manuscript by Upadhyay et al is a protocol for cloning, expression and purification of the LysE exporter protein of Mycobacterium tuberculosis, an important target used in BCG vaccine. The protocol is well written with sufficient details provided. The reviewer has the following minor suggestions:

 1. Abstract

 - Methods: Change "Amplied" to "Amplified"

 2. Page 3: Methods

 - Instruments required: Change "electrophoretic assembly" to "electrophoresis assembly"

 - Reagents required: remove an extra beginning parenthesis from the front of DH5a

 3. Page 4: Protocol

 Genomic DNA isolation: First sentence is incomplete. Change it to "Isolation and purification of genomic DNA using mycobacterium tuberculosis was performed according to the earlier protocols"

 Page 8: For insoluble protein, 4th line

 4. Replace "clear supernatant" with "clarified supernatant"

 5. Page 9: Chromatographic conditions:

 Change the 5th sentence to:

 Protocol may need to be changed according to protein behavior during purification and stability.

Is the study design appropriate for the research question?

Yes

Is the rationale for, and objectives of, the study clearly described?

Yes

Are sufficient details of the methods provided to allow replication by others?

Yes

Are the datasets clearly presented in a useable and accessible format?

Not applicable

Reviewer Expertise:

Autoimmunity

I confirm that I have read this submission and believe that I have an appropriate level of expertise to confirm that it is of an acceptable scientific standard.

# Reviewer report: Reviewer response for version 2

**DOI:** 10.5256/f1000research.150461.r176987

**Published:** 2023-07-19

**Authors:** Seamus J. Martin, Seren Hamsici

**Affiliations:** 1 Molecular Cell Biology Laboratory, Department of Genetics, The Smurfit Institute, Trinity College Dublin, Dublin, Ireland; 2 Dept. of Genetics, The University of Dublin Trinity College, Dublin, Leinster, Ireland

I am happy with the revisions.

Is the study design appropriate for the research question?

Yes

Is the rationale for, and objectives of, the study clearly described?

Yes

Are sufficient details of the methods provided to allow replication by others?

Yes

Are the datasets clearly presented in a useable and accessible format?

Not applicable

Reviewer Expertise:

Immunology, cell biology, inflammation, protein expression, protein purification,

We confirm that we have read this submission and believe that we have an appropriate level of expertise to confirm that it is of an acceptable scientific standard.

# Reviewer report: Reviewer response for version 1

**DOI:** 10.5256/f1000research.144639.r167046

**Published:** 2023-05-26

**Authors:** Seamus J. Martin, Seren Hamsici

**Affiliations:** 1 Molecular Cell Biology Laboratory, Department of Genetics, The Smurfit Institute, Trinity College Dublin, Dublin, Ireland; 2 Dept. of Genetics, The University of Dublin Trinity College, Dublin, Leinster, Ireland

In this study protocol, the authors report a protocol for the purification of lysine exporter (LysE) protein, which is a potential drug target, vaccine candidate and diagnostic marker for bacterial infections. To study and utilize the protein effectively, it is necessary to obtain a pure and high concentration sample. This protocol provides a method for purifying LysE protein with >95% purity using immobilized metal affinity chromatography (IMAC). The protocol can serve as a useful resource for researchers who study LysE and other transmembrane protein using IMAC.

 The authors are in line with the requirements for study protocol. However, I have few suggestions that can further improve the clarity and reproducibility of the protocol.

  

1. Include information on the quality control of the purified protein: I recommend including more information on the expected yield and purity of the final protein. The article mentions that the purified protein has >95% purity but does not provide any information on yield (how much protein was purified from what volume of bacterial culture?) and the purified protein workflow should be demonstrated on SDS-PAGE gels.
2. The authors should consider adding some subheadings to break up the text into more manageable sections. This would make it easier for the reader to follow the protocol and locate specific steps if they need to refer back to these later.
3. The authors should consider providing some troubleshooting tips for common issues that may occur during the purifications process, especially while purifying the insoluble protein.

Is the study design appropriate for the research question?

Yes

Is the rationale for, and objectives of, the study clearly described?

Yes

Are sufficient details of the methods provided to allow replication by others?

Yes

Are the datasets clearly presented in a useable and accessible format?

Not applicable

Reviewer Expertise:

Immunology, cell biology, inflammation, protein expression, protein purification,

We confirm that we have read this submission and believe that we have an appropriate level of expertise to confirm that it is of an acceptable scientific standard, however we have significant reservations, as outlined above.

# Response

**Published:** 2023-05-29

**Authors:** Shilpa Upadhyay

**Affiliations:** Datta Meghe Institute of Higher Education and Research, India

1. **Comment 1: **In this study protocol, the authors report a protocol for the purification of lysine exporter (LysE) protein, which is a potential drug target, vaccine candidate and diagnostic marker for bacterial infections. To study and utilize the protein effectively, it is necessary to obtain a pure and high concentration sample. This protocol provides a method for purifying LysE protein with >95% purity using immobilized metal affinity chromatography (IMAC). The protocol can serve as a useful resource for researchers who study LysE and other transmembrane protein using IMAC.
**Response 1: **Thank you for appreciating our efforts.
2. **Comment 2: **Include information on the quality control of the purified protein: I recommend including more information on the expected yield and purity of the final protein. The article mentions that the purified protein has >95% purity but does not provide any information on yield (how much protein was purified from what volume of bacterial culture?) and the purified protein workflow should be demonstrated on SDS-PAGE gels.
**Response 2: **Thank you for your comments. We will definitely include this suggestion in the future publications of this work because this study is still ongoing and we are now validating the cloning part. We will definitely include the SDS-PAGE gels picture in further publications.
  In this protocol, we had mentioned that “Similar protocols have already been adapted to purify other mycobacterial proteins in our collaborative lab with >95% purity” under the supervision of protein experts.
  Although, from 1 L culture, we were able to purify 10 -15 mg of similar type of mycobacterial protein. This yield depends upon:
  1. The expression of protein depending on type of construct, amino acids sequence, extra tags, folding nature of protein, expression time, temperature and concentration of IPTG. However, only not limited to mention.
  2. To purify protein with high purity (>95%), we are adopting the following steps. Which we will publish in details later on getting recombinant protein.
  First step: As we made the construct in such a way so that protein yield would get as much as at increased level.
  Second step: The interacting proteins from protein expressing strain with target protein should be as low as possible. However, placing 6 His tag with target protein minimized the problem of protein binding column.
  Third step: buffers and their stock should be prepared in autoclaved water and stored after filtration.
  3. High yield of protein also depends upon the lab conditions. In our Lab, we are following the Good Laboratory Practice (GLP).
3. **Comment 3:** The authors should consider adding some subheadings to break up the text into more manageable sections. This would make it easier for the reader to follow the protocol and locate specific steps if they need to refer back to these later.
**Response 3:** Thank you for your comments. We will definitely incorporate your suggestion.
4. **Comment 4:** The authors should consider providing some troubleshooting tips for common issues that may occur during the purifications process, especially while purifying the insoluble protein.
**Response 4: **Thank you for your comments. We will definitely incorporate your suggestion
  Troubleshooting tips:
  1. During Ni-NTA purification, sometimes, while adding the supernatant containing extraction buffer (50 m MTris-HCl pH 8.0, 100-300 mM NaCl, 10-20% glycerol, 10 mM βME), the color of the resin may change from blue to brown. This is due to the presence of β-mercaptoethanol (reducing agent) in extraction buffer. This brown color of resin may disappear after 3-4 washing with wash buffer A containing no βME. This color change will not affect the purification step.
  2. During dialysis or refolding of insoluble protein, sometimes protein may get precipitate. To avoid the precipitation, step wise dialysis is required. Since the elution buffer for insoluble protein contains 6M or 8M urea. Step wise removal of urea is recommended. For e.g., use dialysis buffer 1 with 4M urea, dialysis buffer 2 with 2 M urea, dialysis buffer 3 with 1M urea and then the final dialysis buffer with no urea.
  3. Concentration of Urea (6M to 8M) may change depending of the nature of protein.
  This is a protocol and I have added in **Notes **that “While performing experiments, the buffer’s composition and experimental conditions may slightly differ from the composition and conditions mentioned above depending on the protein's behavior and stability (secretory or insoluble)”.
